# Differences in the accumulation of phosphorus between vegetative cells and heterocysts in the cyanobacterium *Nodularia spumigena*

**DOI:** 10.1038/s41598-018-23992-1

**Published:** 2018-04-04

**Authors:** Philipp D. Braun, Heide N. Schulz-Vogt, Angela Vogts, Monika Nausch

**Affiliations:** Leibniz Institute for Baltic Sea Research Warnemünde, Department of Biological Oceanography, Rostock, 18119 Germany

## Abstract

The cyanobacterium *Nodularia spumigena* is a species that frequently forms blooms in the Baltic Sea. Accumulation of the vital nutrient phosphorus (P) apparently plays an important role in the ability of this and other cyanobacteria to grow even when dissolved inorganic phosphorus is depleted. However, until now, this has not been studied in *N. spumigena* at the cellular level. Therefore, in this study, phosphorus incorporation and distribution in cyanobacterial filaments over time was examined by scanning electron microscopy in combination with energy dispersive X-ray analysis (SEM/EDX) and nanoscale secondary ion mass spectrometry (NanoSIMS). Immediately after phosphate addition to a phosphorus-depleted population, the phosphate concentration decreased in the water while intracellular polyphosphate accumulated. Microscopically, phosphorus in form of polyphosphate granules was stored preferentially in vegetative cells, whereas heterocysts remained low in intracellular phosphorus. This information is an essential step towards understanding the phosphorus dynamics of this species and demonstrates that the division of tasks between vegetative cells and heterocysts is not restricted to nitrogen fixation.

## Introduction

**Figure 1:**
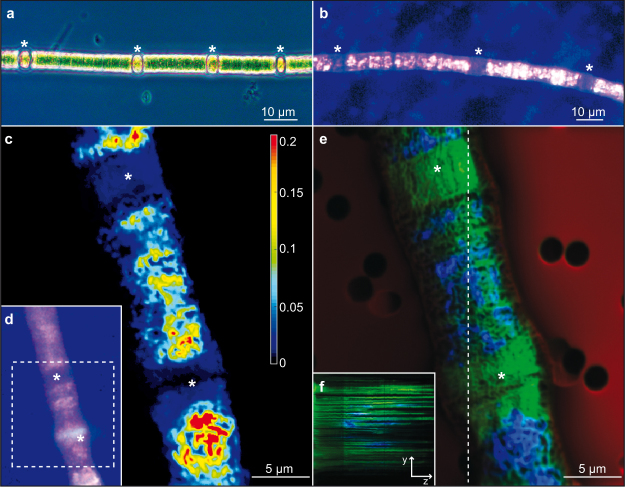
Micrographs of *N*. *spumigena* filaments taken with different microscopic techniques. (**a**) Light microscopy. (**b**) Fluorescence microscopy; Filament was stained with DAPI; Polyphosphate inclusions are visible as bright yellow/white dots. (**c** and **e**) NanoSIMS images; The investigated filament segment is marked in the fluorescence micrograph (**d**) (broken line). (**c**) The intensity of ^31^P^−^/^12^C^−^ is shown. (**e**) Overlay picture of ^12^C^−^ (red; filter background), ^12^C^14^N^−^ (green, representative of organic material) and ^31^P^−^ (blue); Broken line marks the y-axis of the merged stack through the filament in inserted picture (**f**). (**f**) Shows the distribution of ^31^P^−^ (blue) and ^12^C^14^N^−^ (green) through the filament width (z-axis). A“*” in all pictures marks a heterocyst.

In surface waters of marine and freshwater ecosystems, phosphorus availability can limit phytoplankton^1^ and bacterial growth^2^. Diazotrophic cyanobacteria are especially dependent on the availability of phosphorus, because they have the capability to convert dinitrogen to biologically available forms of nitrogen. Therefore, they have an advantage over other phytoplankton and become dominant in the Baltic Sea after the spring bloom, when the N:P ratio of dissolved inorganic nutrients in the water is low^3^. In *Nodularia spumigena* and several other filamentous cyanobacteria, nitrogen fixation takes place in specialized cells called heterocysts that differentiate from vegetative cells when other nitrogen sources, such as ammonium and nitrate, are absent^4–6^. Heterocysts have a unique morphology and are therefore visible by common light microscopy (Fig. 1a).

In the Baltic Sea, the cyanobacterium *N. spumigena* together with *Aphanizomenon* spec. and *Dolichospermum* spec. can form large blooms during the summer months when high temperatures (>16 °C) and sunlight irradiation promotes growth^7–9^. These cyanobacteria contribute up to 1/3 of the annual input of nitrogen to the Baltic Sea^10^. Besides this they can produce toxins^11^, which make them harmful for mammals and birds. Furthermore, decaying cyanobacteria blooms contribute to the acceleration of oxygen depletion in deep basins and the formation of anoxic regions.

The cyanobacteria blooms are highly dependent on the availability of phosphorus^12^. As many other cyanobacteria, *N. spumigena* is able to take up phosphate and accumulate it intracellularly. This stored phosphate is later used under conditions of phosphorus deficiency to support cell metabolism and growth^12^. However, it is still unknown how this excess phosphate is distributed within the filaments. Therefore, we added phosphate to phosphorus-starved *N. spumigena* and followed the accumulation and distribution of phosphorus in filaments over time by using scanning electron microscopy in combination with energy dispersive X-ray analysis (SEM/EDX) and nanoscale secondary ion mass spectrometry (NanoSIMS).

## Results

**Figure 2:**
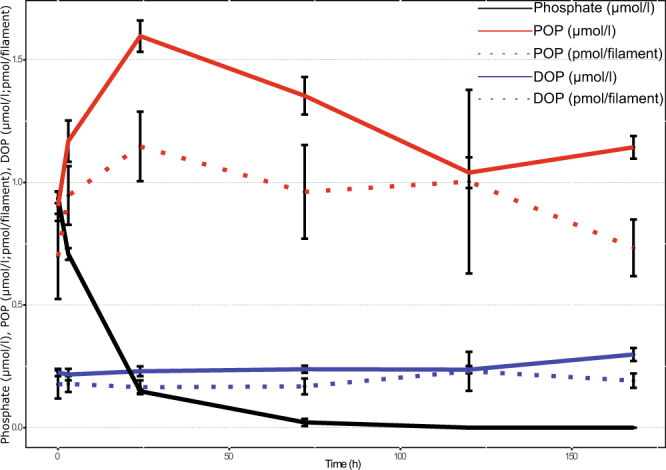
Changes in phosphorus fractions during the experiment. Particulate organic phosphorus (POP) and dissolved organic phosphorus (DOP) are given in µmol/l (continuous line) and pmol/filament (dotted line). Phosphate concentration in the water is expressed in µmol/l.

After the addition of phosphate to the phosphorus-starved *N. spumigena*, the phosphate concentration in the ambient water decreased rapidly (Fig. 2). After three to five days, the phosphate was completely consumed and the particulate (cyanobacterial) organic phosphorus increased. Dissolved organic phosphorous concentrations remained unchanged over the experiment (Fig. 2).

### Light-, fluorescence microscopy and NanoSIMS

4′6-diamidino-2-phenylindole (DAPI)^13^ stained filaments from the first three days (0, 24 and 72 h), during which most of the phosphorus uptake occurred, gave a first impression of the overall distribution of phosphorus (Fig. 1b and d). Even at this low magnification, it became apparent that heterocysts seemed to contain no polyphosphate granules when compared to the vegetative cells (Fig. 1b). Nevertheless, as staining is only an indirect indication and observation of the polyphosphate granules could be hindered, e.g. by thicker cell walls, we verified this finding by NanoSIMS analyses. To date NanoSIMS analyses were performed in a very few studies addressing nutrient uptake of cyanobacteria^14,15^. In this study we analysed eight filaments (n = 20 measurements in total) after one and five days of phosphorus uptake. The ^31^P/^12^C^−^ (Fig. 1c) and ^31^P^−^/^12^C^14^N^−^ (Supplementary Fig. 1b) ratios over the filaments confirmed a very low phosphorus content in the heterocysts compared to vegetative cells (Fig. 1c). Single channel pictures of ^31^P^−^ showed phosphorus everywhere in the filament, but the signal was very weak in the heterocysts. An overlay picture of ^12^C^−^, ^12^C^14^N^−^ and ^31^P^−^ showed a strong phosphorus signal only in the vegetative cells (Fig. 1d). In a depth scan, we observed that the phosphorus signal started to increase further inside the filaments compared to the nitrogen signal, which was distributed more evenly (Fig. 1f).

### SEM

**Figure 3:**
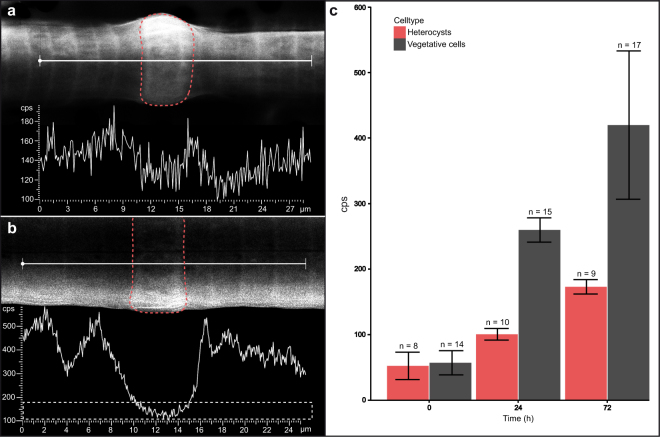
Phosphorus accumulation in vegetative cells and heterocysts in *N. spumigena* during the experiment analysed with SEM. (**a** and **b**) SEM/EDX pictures of the *N. spumigena* filaments at different time points and the corresponding line scans of the phosphorus signal. The signals of phosphorus over the measured line (white line in the filaments) are given in counts s^−1^ (cps); the area marked with a broken red line shows the heterocyst. (**a**) 0 h after beginning of the experiment. (**b**) After 24 h of incubation; the area marked with a broken line shows the range of values at 0 h; in the heterocyst, the values remained low. (**c**) Changes in the phosphorus signal intensity over time in vegetative cells (grey) and heterocysts (red) calculated from the excitation energy peak of phosphorus in point measurements. n = number of measurements in four (0 h) and three (each 24 h and 72 h) filaments.

In order to follow the increase of intracellular phosphorus over time, we performed line scans on nine filaments (n = 10 measurements in total) as well as point measurements on ten filaments (n = 73 measurements in total) by SEM/EDX. Line scans showed that initially under phosphorus-depleted conditions, the phosphorus signals in both vegetative cells and heterocysts were in the range of 110–180 counts s^−1^ (Fig. 3a). After one day, the phosphorus signal in the vegetative cells was up to three times higher compared to the beginning of the experiment (Fig. 3b) and increased up to 800 counts s^−1^ after three days. In contrast, the phosphorus signal in the heterocyst did not increase after one day and remained in a low range of 110–180 counts s^−1^. After three days, the heterocystous phosphorus content was still only 200 counts s^−1^. A larger number of point measurements (Fig. 3c; element analysis in Supplementary Fig. 1a) supported this general pattern of phosphorus uptake. Here the phosphorus values in the vegetative cells were shown to be five times higher after one day and up to nine times higher after three days. Again, the heterocystous phosphorus content changed less, although a slight increase in phosphorus was detectable, even though an accumulation in the form of granules was not observed (Fig. 3b and c). After one day, the phosphorus content doubled, and after three days, it was up to four times higher. These differences in phosphorus content between cell types and between time points were statistically significant (ANOVA: vegetative cells: *F* = 101.3, *p* < 0.0001; heterocysts: *F* = 160.7, *p* < 0.0001; Tables 1 and 2).

**Table 1:** T-Test between vegetative cells and heterocysts at different times.

	Vegetative Cells				Heterocysts				*t value*	*p value*
	Mean (cps)	SD (cps)	n	d. f.	Mean (cps)	SD (cps)	n	d. f.		
0 h	57	19	14	13	52	21	8	7	0.55	0.59
24 h	260	19	15	14	101	9	10	9	25.20	<0.0001
72 h	420	113	17	16	173	11	9	8	6.46	<0.0001

Values were calculated from the excitation energy peak of phosphorus in point measurements. SD = Standard Deviation; n = number of measurements in four (0 h) and three (at 24 h and 72 h) filaments; d. f. = degrees of freedom; cps = counts s^−1^.

**Table 2:** ANOVA to test the statistically significant differences in the phosphorus content of vegetative cells and heterocysts at different time points.

	Source of Variation	Sum of Squares	d. f.	Mean Squares	*F value*	*p value*
Vegetative Cells	Between Groups	1.01E + 06	2	5.05E + 05	101.3	<0.0001
	Within Groups	2.14E + 05	43	4.99E + 03		
	Total	1.22E + 06	45			
Heterocysts	Between Groups	6.30E + 04	2	3.15E + 04	160.7	<0.0001
	Within Groups	4.71E + 03	24	1.96E + 02		
	Total	6.77E + 04	26			

d. f. = degrees of freedom.

## Discussion

The pronounced difference in phosphorus accumulation between two neighbouring cells of a filament clearly demonstrates that polyphosphate formation is a specific physiological reaction of vegetative cells to the environmental conditions. Since heterocysts differentiated from vegetative cells^5,16^, they can be assumed to have the genes required for polyphosphate accumulation, but their physiological state seems to prevent polyphosphate formation. Even though we can currently only speculate on the reasons for this observation, it is very important for the interpretation of polyphosphate accumulation in general. Most studies that quantify polyphosphate, e.g. in the oligotrophic oceans^1,17,18^, have not identified the organisms responsible for the observed polyphosphate distribution. However, even if these organisms were known, judging by our findings it would be necessary to also know the current metabolism of the cells to understand the underlying mechanisms.

Our result clearly shows differences in the accumulation of phosphorus in the different cell types, but at the beginning of the experiment, the amount of phosphorus did not differ between them. There seem to be a basic amount of phosphorus in both cell types, probably in form of DNA, RNA, phosphorus lipids, ATP, NADPH, etc., but polyphosphate inclusions are exclusively observed in vegetative cells (Fig. 3b). Theoretically, there could be numerous reasons why the heterocyst did not form polyphosphate granules. As they do not divide, they may not need to accumulate extra phosphorus for later growth. In addition, heterocysts have the task to fix nitrogen and the nitrogenase needs to be protected from oxygen. For this reason, heterocysts have unusual cell walls, which are thicker and contain glycol- and phospholipids and a polysaccharide layer^19,20^. Therefore, the slight increase in the phosphorus content could be due to the build-up of thicker cell walls, and the storage of phosphoric lipids into them.

Another explanation for the slight increase could be the need for energy to maintain the nitrogenase activity. Energy in form of ATP and NADPH is obtained from carbohydrates (sucrose) supplied by the neighbouring cells and in turn the heterocysts export fixed nitrogen in form of glutamine and other amino acids^21,22^. According to Haselkorn^16^ and Böhme^21^, within the heterocyst glucose-6-phosphate is transferred to 6-phosphogluconic acid and then ribulose-5-phosphate. This process provides two molecules of NADPH, which are used to reduce ferredoxin. The transport mechanism of maltose into the heterocyst as well as the following reduction processes to glucose-6-phosphate are still unknown. However, our data do not reveal whether the process of phosphorus incorporation into the heterocyst is supported by an active transport through the microplasmodesmata or just by diffusion. The need of ATP, which is provided by the photosystem I, and of NADPH to maintain nitrogen fixation, could explain why we found a slight increase of phosphorus in the heterocysts as well. Due to the loss of photosystem II, heterocyst have a much lower CO_2_ fixing capability compared to vegetative cells^23^, as this process would compete with nitrogen fixation for reductant and ATP (reviewed by Haselkorn)^16^. Another reason for the lack of polyphosphate inclusions could be the alkalinisation of the heterocysts due to the forming of ammonium via nitrogen fixation^24,25^, which may cause the hydrolysis of polyphosphates.

It is possible, that all these differences in metabolism between the two cell types together release the heterocyst from numerous stresses experienced by vegetative cells (e.g. radicals, nitrogen or energy limitation, pH, etc.), and therefore they do not need polyphosphate to regulate their response to physiological stress^19,20,24–27^. Whichever reason lies behind these pronounced differences in polyphosphate content, it is clear that the division of tasks between heterocysts and vegetative cells is further evolved than previously believed. To connect the results presented in this study to the global ocean system, more studies on polyphosphate inclusions and storages in different cell types in other heterocystous cyanobacteria, e.g. *Trichodesmium* should be conducted. In addition, future studies should address the question, where and in which form phosphorus is stored in non-filamentous cyanobacteria.

## Methods

### Sample collection

Samples of *N. spumigena* were collected with a 55 µm plankton net from phosphorus-depleted surface waters of the Baltic Sea at 58°53.000′N, 20°19.000′E during cruise M-117 on the research vessel “*Meteor*” in August 2015. In experiments on board phosphate was added to the phosphorus-starved population to a final concentration of 1 µmol/L. The cyanobacteria were incubated for seven days, and subsamples were filtered after 0, 24 and 72 h on 3 µm polycarbonate membrane filters (ø 25 mm) for the microscopic analyses with SEM/EDX and NanoSIMS.

### Light- and fluorescence microscopy

100 ml unfiltered subsamples from the first three days (0, 24 and 72 h), during which most of the phosphorus uptake occurred, were incubated with 300 µl Lugol’s iodine in 300 ml amber bottles. Light microscopy (phase-contrast microscopy) of the filaments was performed by using a Carl Zeiss “Axiovert S100”. Measurements and pictures were taken with “ZENlite” Software from Carl Zeiss Microscopy, Jena.

For the fluorescence microscopy 2 ml subsamples from the first three days (0, 24 and 72 h) were filtrated with a low-pressure of 150 mbar on 3 µm polycarbonate membrane filters (ø 25 mm). Afterwards 1 ml DAPI (1 mg/100 ml) was added on top of the filter and incubated for 5 min. in the dark. Then, DAPI was filtered off and the filter were washed with 1 ml of Milli-Q water. Filters were analysed with a Leica Laser Microdissection microscope (LMD 7000; Leica Camera, Wetzlar) and the related software “LMD V7.6”. This method was used to identify regions of interest for NanoSIMS and SEM/EDX analyses.

### NanoSIMS

SIMS imaging was performed using a NanoSIMS 50 L instrument (Cameca, France) at the Leibniz-Institute for Baltic Sea Research Warnemünde (IOW). A ^133^Cs^+^ primary ion beam was used to erode and ionize atoms of the sample. Among the received secondary ions, images of ^12^C^−^, ^16^O^−^, ^12^C^14^N^−^, ^31^P^−^, and secondary electrons were recorded simultaneously for areas of interest using mass detectors equipped with electron multipliers (Hamamatsu). Mass resolution was 8000 or better (according to the definition of CAMECA).

Prior to the analysis, sample areas of 60*60 µm were sputtered for 200 s with 600 pA to reach the steady state of secondary ion formation, erode the gold and clean the surface. In a first step, the area was depicted using a 1 pA primary ion beam scanning 512*512 px, 0.25 ms/px. After this first analysis, the cyanobacteria were barely eroded, and in order to penetrate to a greater depth in a faster time, the subsequent analyses were performed with a 20 pA primary ion beam on a 35*35 µm raster, scanning 512*512 px, 2 ms/px. After 410 planes, the cyanobacteria of approx. 8 µm diameter were completely consumed. Assuming an ideal cylindrical form of the cyanobacteria, this resulted in an estimated consumption of 20 nm per NanoSIMS plane. Of course, the prior implantation and analysis with a weaker beam had already consumed material, and the cyanobacteria might have had a compressed ellipsoid cross section. Thus, the real erosion might be lower, but one should note that material is mixed in the surface layer by the penetrating ion beam, which leads to ion contributions of a few additional nm. Of the 410 stack plane, 106 to 245 were used to depict the phosphorus distribution (Fig. 1c and e). For data analysis, the Look@NanoSIMS software^28^ was employed.

### SEM

SEM analysis was performed using a “Zeiss MERLIN VP compact” at the Leibniz-Institute for Baltic Sea Research Warnemünde (IOW). Measurements were performed with the “AZtecEnergy“ program (Oxford Instruments). Measurements were done under high vacuum conditions with an acceleration voltage of 15 kV. The measured values are not corrected X-Ray events and were measured in counts s^−1^ at the excitation energy peak of phosphorus at 2.0134 keV. Before analyzing, the samples were coated with chrome to prevent wavelength interference of phosphorus with the signal of gold, which is usually used for coating.

### Statistical analyses

Two two-tailed statistical methods were used. The t-test was used to find significant differences between vegetative cells and heterocysts at the same time of each sample taking points (Table 1). The measured values were calculated from the excitation energy peak of phosphorus in point measurements. To test the significant differences in the phosphorus content of vegetative cells and heterocysts at different time points a ANOVA was performed (Table 2; http://www.physics.csbsju.edu/stats/anova.html).

### Data availability

The data sets generated and analysed during the current study are available from the corresponding author on reasonable request.

## Electronic supplementary material

Supplementary Figure 1

## References

[1] Diaz JM (2012). Potential role of inorganic polyphosphate in the cycling of phosphorus within the hypoxic water column of Effingham Inlet, British Columbia. Global Biogeochem. Cycles.

[2] Karl DMP (2000). the staff of life. Nature.

[3] Janssen F, Neumann T, Schmidt M (2004). Inter-annual variability in cyanobacteria blooms in the Baltic Sea controlled by wintertime hydrographic conditions. Mar. Ecol. Prog. Ser..

[4] Meeks JC, Elhai J (2002). Regulation of Cellular Differentiation in Filamentous Cyanobacteria in Free-Living and Plant-Associated Symbiotic Growth States. Microbiol. Mol. Biol. Rev..

[5] Wolk, C. P., Ernst, A. & Elhai, J. Heterocyst Metabolism and Development. In *The Molecular Biology of Cyanobacteria*. *Advances in**Photosynthesis* (ed. Bryant, D. A.) **1**, 769–823 (Springer, 2004).

[6] Zhang C-C, Laurent S, Sakr S, Peng L, Bédu S (2006). Heterocyst differentiation and pattern formation in cyanobacteria: a chorus of signals. Mol. Microbiol..

[7] Wasmund N (1997). Occurrence of Cyanobacterial Blooms in the Baltic Sea in relation to Environmental Conditions. Int. Rev. der gesamten Hydrobiol. und Hydrogr..

[8] Nausch M, Nausch G, Wasmund N (2004). Phosphorus dynamics during the transition from nitrogen to phosphate limitation in the central Baltic Sea. Mar. Ecol. Prog. Ser..

[9] Degerholm J, Gundersen K, Bergman B, Söderbäck E (2006). Phosphorus-limited growth dynamics in two Baltic Sea cyanobacteria, *Nodularia* sp. and *Aphanizomenon* sp. FEMS Microbiol. Ecol..

[10] Wasmund N, Nausch G, Schneider B, Nagel K, Voss M (2005). Comparison of nitrogen fixation rates determined with different methods: a study in the Baltic Proper. Mar. Ecol. Prog. Ser..

[11] Luckas B (2005). Overview of key phytoplankton toxins and their recent occurrence in the North and Baltic Seas. Environ. Toxicol..

[12] Nausch M (2009). Phosphorus input by upwelling in the eastern Gotland Basin (Baltic Sea) in summer and its effects on filamentous cyanobacteria. Estuar. Coast. Shelf Sci..

[13] Hupfer M, Gloess S, Grossart H-P (2007). Polyphosphate-accumulating microorganisms in aquatic sediments. Aquatic Microbial Ecology.

[14] Ploug H (2011). Carbon, nitrogen and O_2_ fluxes associated with the cyanobacterium *Nodularia spumigena* in the Baltic Sea. ISME J..

[15] Sukenik A (2015). Carbon assimilation and accumulation of cyanophycin during the development of dormant cells (akinetes) in the cyanobacterium *Aphanizomenon ovalisporum*. Front. Microbiol..

[16] Haselkorn R (1978). Heterocysts. Annu. Rev. Plant Physiol..

[17] Diaz JM (2008). Marine Polyphosphate: A Key Player in Geologic Phosphorus Sequestration. Science.

[18] Martin P, Van Mooy BAS (2013). Fluorometric Quantification of Polyphosphate in Environmental Plankton Samples: Extraction Protocols, Matrix Effects, and Nucleic Acid Interference. Appl. Environ. Microbiol..

[19] Ernst A (1992). Synthesis of Nitrogenase in Mutants of the Cyanobacterium *Anabaena* sp. Strain PCC 7120 Affected in Heterocyst Development or Metabolism. J. Bacteriol..

[20] Soriente A, Sodano G, Cambacorta A, Trincone A (1992). Structure of the ‘Heterocyst Glycolipids’ of the marine Cyanobacterium *Nodularia harveyana*. Tetrahedron.

[21] Böhme H (1998). Regulation of nitrogen fixation in heterocyst-forming cyanobacteria. Trends Plant Sci..

[22] Golden JW, Yoon H-S (2003). Heterocyst development in. Anabaena. Curr. Opin. Microbiol..

[23] Fay P, Walsby AE (1966). Metabolic Activities of Isolated Heterocysts of the Blue-green Alga *Anabaena cylindrica*. Nature.

[24] Pick U, Rental M, Chitlaru E, Weiss M (1990). Polyphosphate-hydrolysis - a protective mechanism against alkaline stress?. FEBS Lett..

[25] Pick U, Weiss M (1991). Polyphosphate Hydrolysis within Acidic Vacuoles in Response to Amine-Induced Alkaline Stress in the Halotolerant Alga *Dunaliella salina*. Plant Physiol..

[26] Fay P (1992). Oxygen Relations of Nitrogen Fixation in Cyanobacteria. Microbiological Reviews.

[27] Kuroda A, Ohtake H (2000). Molecular analysis of polyphosphate accumulation in bacteria. Biochem. Biokhimiia..

[28] Polerecky L (2012). Look@NanoSIMS - a tool for the analysis of nanoSIMS data in environmental microbiology. Environ. Microbiol..

