# Supplementary material for: Differences in the accumulation of phosphorus between vegetative cells and heterocysts in the cyanobacterium Nodularia spumigena
Source: Sci Rep. 2018 Apr 4;8:5651. doi: 10.1038/s41598-018-23992-1 (PMC5884831; doi:10.1038/s41598-018-23992-1)
Supplement: Supplementary file 1 — Supplementary Figure 1 [file 41598_2018_23992_MOESM1_ESM.pdf]

Supplementary information

Title: "Differences in the accumulation of phosphorus between vegetative cells and heterocysts in the cyanobacterium *Nodularia spumigena*"

Authors: Philipp D. Braun, Heide N. Schulz-Vogt, Angela Vogts, Monika Nausch

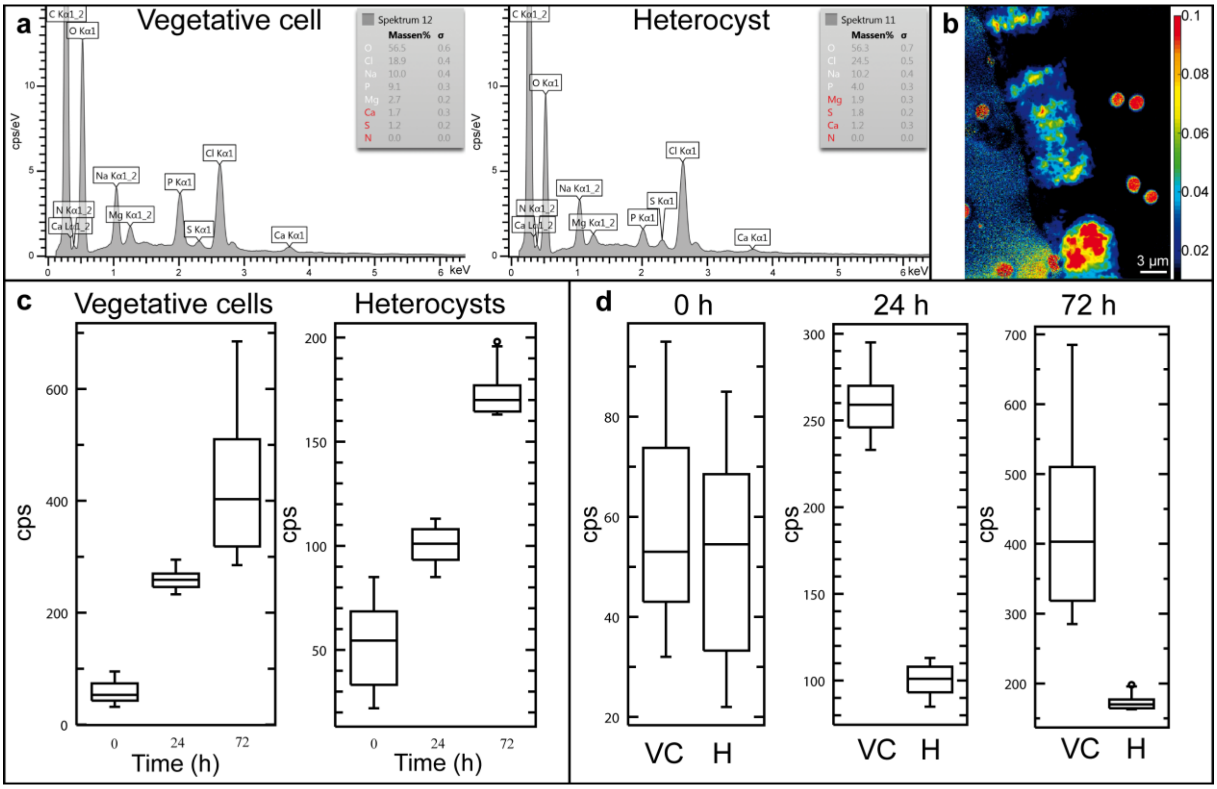

Supplementary Figure 1. The additional measured parameters of the study are shown as well as the plots from the statistical analyses and a NanoSIMS image. **(a)** Example of SEM/EDX spectra from a measurement in a vegetative cell and in a heterocyst. **(b)** NanoSIMS image of the $^{31}P^{-}/^{12}C^{14}N^{-}$ ratio. **(c)** ANOVA between time points in vegetative cells and heterocysts. **(d)** T-test between vegetative cells (VC) and heterocysts (H) at three incubation periods.

cps = counts s^{-1}
